# Psychometric properties of a single-item scale to assess sleep quality among individuals with fibromyalgia

**DOI:** 10.1186/1477-7525-7-54

**Published:** 2009-06-17

**Authors:** Joseph C Cappelleri, Andrew G Bushmakin, Anne M McDermott, Alesia B Sadosky, Charles D Petrie, Susan Martin

**Affiliations:** 1 Pfizer Inc, Global Research and Development, 50 Pequot Avenue, New London, Connecticut 06320, USA; 2 Outcomes Research Consultant, 13104 Riviera Terrace, Silver Spring, Maryland 20904, USA; 3 Pfizer Inc., Global Outcomes Research, 235 East 42nd Street, New York, New York 10017, USA; 4 Pfizer Inc, Global Outcomes Research, 2800 Plymouth Road, Ann Arbor, Michigan 48105, USA

## Abstract

**Background:**

Sleep disturbances are a common and bothersome symptom of fibromyalgia (FM). This study reports psychometric properties of a single-item scale to assess sleep quality among individuals with FM.

**Methods:**

Analyses were based on data from two randomized, double-blind, placebo-controlled trials of pregabalin (studies 1056 and 1077). In a daily diary, patients reported the quality of their sleep on a numeric rating scale ranging from 0 ("best possible sleep") to 10 ("worst possible sleep"). Test re-test reliability of the Sleep Quality Scale was evaluated by computing intraclass correlation coefficients. Pearson correlation coefficients were computed between baseline Sleep Quality scores and baseline pain diary and Medical Outcomes Study (MOS) Sleep scores. Responsiveness to treatment was evaluated by standardized effect sizes computed as the difference between least squares mean changes in Sleep Quality scores in the pregabalin and placebo groups divided by the standard deviation of Sleep Quality scores across all patients at baseline.

**Results:**

Studies 1056 and 1077 included 748 and 745 patients, respectively. Most patients were female (study 1056: 94.4%; study 1077: 94.5%) and white (study 1056: 90.2%; study 1077: 91.0%). Mean ages were 48.8 years (study 1056) and 50.1 years (study 1077). Test re-test reliability coefficients of the Sleep Quality Scale were 0.91 and 0.90 in the 1056 and 1077 studies, respectively. Pearson correlation coefficients between baseline Sleep Quality scores and baseline pain diary scores were 0.64 (p < 0.001) and 0.58 (p < 0.001) in the 1056 and 1077 studies, respectively. Correlations between the Sleep Quality Scale and the MOS Sleep subscales were statistically significant (p < 0.01), except for the MOS Snoring subscale. Across both studies, standardized effect sizes were generally moderate (0.46 to 0.52) for the 300 mg group and moderate (0.59) or moderate-to-large (0.70) for the 450 mg group. In study 1056, the effect size for the 600 mg group was moderate-to-large (0.73). In study 1077, the effect size for the 600 mg group was large (0.82).

**Conclusion:**

These results provide evidence of the reproducibility, convergent validity, and responsiveness to treatment of the Sleep Quality Scale and provide a foundation for its further use and evaluation in FM patients.

## Background

The American College of Rheumatology (ACR) defines fibromyalgia syndrome (FM) using two criteria: (1) chronic widespread pain and (2) pain upon digital palpation in at least 11 of 18 defined tender point sites [1,2]. Common co-morbid symptoms associated with FM include sleep disturbances, fatigue, morning stiffness, affective disorders, chronic daily headache, dyscognition, irritable bowel syndrome, and irritable bladder [3]. In a series of focus group and ranking exercises, clinical experts and patients agreed that while pain is the cardinal symptom of FM, it is important to also assess fatigue, impact on sleep, health-related quality of life, depression, and cognitive difficulties [4]. Assessing the effectiveness of new therapies therefore requires accurate assessment of a multi-dimensional array of symptoms and problems.

This paper focuses on the measurement of sleep problems in patients with FM. Disturbed sleep is consistently ranked by patients as a highly bothersome symptom of the disease [5,6]. While FM patients report that pain interferes with their sleep, recent studies also suggest a reciprocal relationship. Specifically, sleep quality is predictive of pain as well as broader areas of functioning and emotional well-being such as fatigue, social functioning, and depression [7,8].

While multiple-item, patient-reported surveys are available to measure specific sleep problems, single-item assessments of overall sleep quality provide a useful summary measure and are frequently included in research on FM [9]. Single-item sleep quality assessments allow patients to implicitly weight the various components of sleep that are important to them and assign an overall rating based on their individual rankings and experiences. While rankings and experiences will vary, it is likely that there is a common set of key components that constitute overall sleep quality. A recent study of the subjective meaning of sleep quality among individuals with insomnia and normal sleepers found that both groups similarly defined sleep quality as tiredness on waking and throughout the day, feeling rested and restored on waking, and the number of awakenings experienced in the night [10].

Single-item sleep quality assessments are practical when measurements are taken at frequent intervals, such as in patient diaries that are completed every day. Sleep diaries are reliable and valid assessments for capturing such patient-reported outcomes and reduce recall bias since they are collected on a daily basis [11]. This study reports psychometric properties of a single-item Sleep Quality numeric rating scale completed by FM patients daily in two clinical trials.

## Methods

### Study design and subjects

This paper reports the psychometric properties of the daily diary Sleep Quality Scale using data from two randomized, double-blind, placebo-controlled clinical trials of pregabalin conducted in the United States (US), referred to here as studies 1056 [12] and 1077 [13]. The study designs for these trials have been described elsewhere [12,13]. The studies were randomized, double-blind, and placebo-controlled clinical trials of three doses of pregabalin (300 mg/day, 450 mg/day, and 600 mg/day). Patients were 18 years of age or older with FM as defined by the ACR criteria [1,2].

During the baseline phase, study patients had to have an average daily diary pain score of at least 4 (within the last 7 days) on a numeric rating scale ranging from 0 ("no pain") to 10 ("worst possible pain"). Further, study patients had to have a score of at least 40 mm on the 100 mm visual analogue scale (VAS) of the Short-Form McGill Pain Questionnaire [14] at the screening and baseline (randomization) study visits. In study 1077, patients with a 30% or greater reduction in the VAS from the screening to the randomization study visits (a single-blind placebo run-in period) were discontinued; this criterion in study 1077 was intended to exclude potential placebo responders. In both studies, the primary efficacy measure was endpoint mean pain score defined as the mean of the last 7 pain diary entries while the patient was on study medication.

### The Sleep Quality Scale

In the daily diary assessment, patients reported the quality of their sleep over the past 24 hours on an 11-point numeric rating scale ranging from 0 ("best possible sleep") to 10 ("worst possible sleep"). Patients were instructed to complete the scale in the morning upon awakening. The baseline scores were computed as the average rating over the 7 days prior to taking study medication. The end of treatment score was computed as the average rating over the last 7 days during which the patient was receiving study medication. Since higher scores indicate poorer sleep, negative change scores (end of treatment score minus baseline score) indicate improvement.

### The MOS Sleep Scale

The studies also included a validated sleep survey, the MOS Sleep Scale [15-17]. The MOS Sleep Scale provided 6 subscale scores (Sleep Disturbance, Snoring, Awakening Short of Breath or with a Headache, Quantity of Sleep, Sleep Adequacy, and Somnolence) and an overall Sleep Problems Index score [15]. The Quantity of Sleep subscale score documented the number of hours of sleep per night (possible range from 0 to 24 hours). The remaining scale scores ranged from 0 to 100 where higher scores indicated greater sleep dysfunction, except for the Sleep Adequacy subscale where higher scores reflected more adequate sleep.

### Statistical methods

Test re-test reliability and convergent validity analyses of the Sleep Quality Scale were obtained from all available study patients across all treatment groups. Test re-test reliability of the Sleep Quality scores were evaluated using pre-treatment data. Intraclass correlation coefficients (ICC) based on the daily assessments were computed and the Spearman-Brown Prophecy formula was used to calculate the reliability of the Sleep Quality score (average of 7 daily assessments) [18,19]. Reliability coefficients less than or equal to 0.70 were considered unacceptable; coefficients between 0.70 and 0.90 were considered acceptable; and coefficients of 0.90 or higher were considered excellent levels of test re-test reliability [20].

Convergent validity analyses were evaluated using baseline data. Baseline Sleep Quality scores were correlated with baseline pain diary and baseline MOS Sleep scores using Pearson correlation coefficients.

Treatment effects on the Sleep Quality Scale have been reported previously [12,13]. Treatment effects were based on analysis of covariance (ANCOVA) models of end-of-treatment Sleep Quality scores with treatment and center as factors and corresponding baseline Sleep Quality scores as covariates. The model-estimated least square mean change scores by treatment group were used in these analyses to compute effect sizes. Specifically, standardized effect sizes were computed as the difference between least squares mean changes in Sleep Quality scores in the pregabalin and placebo groups divided by the standard deviation of Sleep Quality scores across all patients at baseline. These effect sizes were interpreted (in absolute value) as follows: trivial (less than or equal to 0.20), small (0.20), moderate (0.50) and large (0.80) [21].

## Results

Studies 1056 and 1077 included 748 and 745 patients, respectively. Most patients were female (94.4% in study 1056 and 94.5% in study 1077) and white (90.2% in study 1056 and 91.0% in study 1077) (Table 1). In study 1056, the mean age of patients was 48.8 years and the average duration of FM was 9 years. In study 1077, the mean age of patients was 50.1 years and the average duration of FM was 10 years. In both studies, baseline mean pain scores were approximately 7 on a scale from 0 ("no pain") to 10 ("worst possible pain").

**Table 1:** Baseline patient characteristics and sleep scores

**Characteristics**	**Study 1056 (N = 748)**		**Study 1077 (N = 745)**	
	**n**	**%**	**n**	**%**
	
**Gender**				
**Male**	42	5.6	41	5.5
**Female**	706	94.4	704	94.5
**Race**				
**White**	675	90.2	678	91.0
**Black**	35	4.7	33	4.4
**Other**	38	5.1	34	4.6
				
	**N**	**Mean ± SD**	**N**	**Mean ± SD**
	
**Age (years)**	748	48.8 ± 10.9	745	50.1 ± 11.4
**Duration of FM Prior to Study Start (months)**	747	111.7 ± 95.0	745	120.2 ± 96.2
**Number of Painful Tender Points^a^**	719	17.1 ± 1.6	723	16.9 ± 1.8
**Baseline Mean Pain Score^b^**	748	7.1 ± 1.3	745	6.7 ± 1.3
**Sleep Quality Scale**^b^	748	6.7 (1.7)	745	6.2 (1.6)
**MOS Sleep Scales**				
**Sleep Disturbance**	744	67.8 ± 23.4	740	60.0 ± 24.9
**Snoring**	726	40.6 ± 35.9	717	36.7 ± 34.6
**Awaken Short of Breath or with Headache**	744	37.6 ± 31.1	743	32.3 ± 32.0
**Quantity of Sleep**	747	5.4 ± 1.6	744	5.6 ± 1.6
**Sleep Adequacy**	745	20.6 ± 22.0	744	23.7 ± 23.2
**Somnolence**	743	50.3 ± 24.1	740	42.1 ± 23.1
**Sleep Problems Index (9-item)**	741	65.0 ± 16.3	736	58.3 ± 17.7

SD = Standard Deviation

^a ^Total number of tender points with value >0 at randomization; the number is missing if any of 18 tender points is missing.

^b ^Baseline = Last 7 available pain scores before taking study medication up to and including Day1. If fewer than 7 scores are available then baseline consists of all scores that are available.

Test re-test reliability coefficients of the pre-treatment Sleep Quality scores, averaged over seven daily measurements prior to study medication, were 0.91 and 0.90 in the 1056 and 1077 studies, respectively (Table 2). Reliability coefficients of this magnitude suggested excellent reproducibility [20].

**Table 2:** Test re-test reliability of pre-treatment Sleep Quality Scale scores

**Study**	**Between-Subjects Error Variance**	**Within-Subject Error Variance**	**ICC of a Single Daily Score**	**ICC for the Average of Seven Daily Scores**
**1056**	2.40	1.68	0.59	0.91

**1077**	2.23	1.69	0.57	0.90

ICC = Intraclass Correlation Coefficient = (Between-Subjects Error Variance)/(Between-Subjects Error Variance + Within-Subject Error Variance)

ICC for the Average of Seven Daily Scores = 7(ICC for single score)/[1 + 6(ICC for a single score)]

Pearson correlation coefficients between baseline Sleep Quality scores and baseline average daily diary pain scores were 0.64 (p < 0.001) and 0.58 (p < 0.001) in the 1056 and 1077 studies, respectively (Table 3). Correlations between the Sleep Quality and the MOS Sleep Scale subscales were statistically significant (p < 0.01), except for the MOS Snoring subscale where no correlation was expected (Table 3). Correlations were largest for the MOS Sleep Disturbance subscale: 0.45 (p < 0.001) and 0.42 (p < 0.001) in studies 1056 and 1077, respectively (Table 3).

**Table 3:** Baseline correlations of the Sleep Quality Scale with pain and the MOS Sleep Scale

	**Pearson Correlation (r) with Sleep Quality Scale (-)**			
	**Study 1056**		**Study 1077**	

	**r**	**p-value**	**r**	**p-value**

**Average Daily Diary Pain Numeric Rating Scale (-)**	0.64	<0.001	0.58	<0.001

**MOS Sleep Scales**				

**Sleep Disturbance (-)**	0.45	<0.001	0.42	<0.001

**Snoring (-)**	0.01	0.884	0.00	0.993

**Awaken Short of Breath or with Headache (-)**	0.21	<0.001	0.14	<0.001

**Quantity of Sleep (+)**	-0.31	<0.001	-0.34	<0.001

**Sleep Adequacy (+)**	-0.21	<0.001	-0.32	<0.001

**Somnolence (-)**	0.11	0.004	0.15	<0.001

(-) Higher scale scores indicate poorer outcome. (+) Higher scale scores indicate better outcome.

As reported previously, all three doses of pregabalin were associated with statistically significantly greater improvements in sleep quality relative to placebo in both the 1056 [12] and 1077 [13] studies. Our current analysis facilitates the interpretation of the magnitude of those treatment differences by reporting effect sizes that were evaluated relative to accepted benchmarks. Across both studies, standardized effect sizes were generally moderate (0.46 to 0.52) for the 300 mg group and moderate (0.59) or moderate-to-large (0.70) for the 450 mg group (Table 4). In study 1056, the effect size for the 600 mg group was moderate-to-large (0.73). In study 1077, the effect size for the 600 mg group was large (0.82).

**Table 4:** Treatment differences and effect sizes for the Sleep Quality Scale

**Pregabalin Dose Group**	**Treatment Comparison** **(Pregabalin – Placebo)^a^**			**Effect Size**^a^
	**Difference**	**95% CI**	**p-value**	

**Study 1056**				

**300 mg**	-0.86	-1.30, -0.43	0.0001	-0.521

**450 mg**	-0.97	-1.40, -0.53	<0.0001	-0.587

**600 mg**	-1.21	-1.64, -0.77	<0.0001	-0.732

**Study 1077**				

**300 mg**	-0.73	-1.14, -0.31	0.0007	-0.458

**450 mg**	-1.12	-1.54, -0.71	<0.0001	-0.703

**600 mg**	-1.31	-1.73, -0.90	<0.0001	-0.822

CI = Confidence Interval

^a^Higher Sleep Quality scores indicate poorer sleep quality. Therefore a negative difference between pregabalin and placebo and negative effect size indicates a greater improvement in sleep quality for patients receiving pregabalin relative to those receiving placebo.

## Discussion

These results suggest that the Sleep Quality Scale has favorable measurement properties. Consistent with the clinical profile of FM, patients' baseline MOS Sleep scores were substantially poorer than the general population values on MOS Sleep scores for Sleep Disturbance (24.5), Snoring (28.3), Awaken Short of Breath or with Headache (9.5), Quantity of Sleep (6.8 hours), Somnolence (21.9), and Sleep Problems Index II (25.8) [16,17]. Test re-test reliability of pre-treatment scores was excellent in both trials. The Sleep Quality Scale was correlated with pain and with relevant aspects of another sleep assessment, the MOS Sleep Scale. Further, the Sleep Quality scale was responsive to treatment effects.

The observed correlations between Sleep Quality scores and pain (0.58 to 0.64) were large and larger than those reported in previous FM studies (0.32 to 0.33) [7,8]. The earlier studies used multi-item sleep quality and pain scales, specifically the Pittsburgh Sleep Quality Index (PSQI) to assess sleep quality and the McGill Pain Questionnaire [8] and SF-36 Bodily Pain scale [7] to assess pain. In the current study, sleep quality and pain were based on single-item, 11-point, numeric rating scales reported by patients in daily diaries.

While these findings support continued applications of the Sleep Quality Scale in FM, we note three areas for further research. First, this study does not address how FM patients define sleep quality. While the p-values for the Pearson correlation coefficient are restricted to the null hypothesis of zero correlation, not to the strength of the correlation, the magnitude of the correlations between the Sleep Quality Scale and the MOS Sleep Scale provides some insights into the specific components of sleep that these patients considered when evaluating the overall quality of their sleep. Specifically, Sleep Quality showed a moderate correlation with MOS Sleep Disturbance, which includes questions about trouble falling asleep, the amount of time to fall asleep, restlessness, and awakening during sleep; a moderate correlation with MOS Quantity of Sleep; a modest correlation with MOS Sleep Adequacy; a small correlation with MOS Somnolence and MOS Awaken Short of Breath or with Headache; and no correlation with MOS Snoring. Qualitative research among FM patients is underway to confirm these findings and to further understand how FM patients evaluate sleep quality.

Second, these clinical trial patients were experiencing fairly high levels of pain and may not be necessarily representative of all FM patient populations; although the patients studied here embody many patients with FM. Consistent with FM being more common in women (3.4%) compared with men (0.5%) [22], the vast majority of patients in this sample were women. Study patients also had a long history of FM, averaging 9 to 10 years. Therefore the clinical and demographic profiles of these samples reflect those mainly of women with about a decade of experience with FM. Applications in real-world settings and within subpopulations of patients, such as children with FM [23], menopausal women with FM [24], and those with newly diagnosed FM [25], would provide additional insight into the impact of FM on sleep quality.

Finally, exploration of frequent comorbid conditions, such as obstructive sleep apnea, and the co-variation of sleep quality and pain merit further study [22,26,27].

## Conclusion

This investigation is a psychometric analysis of a single-item, overall rating of sleep quality for patients with FM. Single-item scales reduce patient burden, particularly when repeated assessments are necessary, such as for ratings recorded in a daily diary. The results of this investigation suggest that the single-item Sleep Quality Scale has favorable measurement properties; namely, these results provide evidence of its reproducibility, convergent validity, and responsiveness to treatment as an overall rating of sleep in two clinical trials. To assess specific areas of sleep, the trials included a multi-item sleep assessment, the MOS Sleep Scale, which also demonstrated favorable psychometric properties in this setting [17]. This paper provides the foundation for further use and evaluation of the Sleep Quality Scale in FM patients, for which sleep disturbances are a key complaint.

## Competing interests

The research reported in this paper was funded by Pfizer Inc. Drs. Cappelleri and Petrie and Mr. Bushmakin are full-time employees of Pfizer Global Research and Development, New London, CT. Dr. Sadosky is a full-time employee of Pfizer Global Pharmaceuticals, Outcomes Research, New York, NY. Ms. Martin, currently at RTI Health Solutions, was a full-time employee of Pfizer Global Research and Development, Outcomes Research, Ann Arbor, MI, when this work was performed. Dr. McDermott was a paid consultant to Pfizer in connection with the development of this manuscript.

## Authors' contributions

JCC and AGB made substantive contributions to the statistical analysis, interpretation of results, and drafting of the manuscript. AM and ABS made substantive contributions in interpreting the analysis and helped to draft the manuscript. SM and CDP made substantive contributions to study design, conduct, and interpretation of results. All authors read and approved the final manuscript.

## References

[B1] Wolfe F, Smythe HA, Yunus MB, Bennett RM, Bombardier C, Goldenberg DL, Tugwell P, Campbell SM, Abeles M, Clark P, Fam AG, Farber SJ, Fletchner JJ, Franklin CM, Gatter RA, Hamaty D, Lessard J, Lichtbroun AS, Masi AT, McCain GA, Reynolds WJ, Romano TJ, Russell IJ, Sheon RP (1990). The American College of Rheumatology 1990 Criteria for the Classification of Fibromyalgia. Report of the Multicenter Criteria Committee. Arthritis Rheum.

[B2] Wolfe F (1993). The epidemiology of fibromyalgia. J Musculoskeletal Pain.

[B3] Wolfe F, Ross K, Anderson J, Russell IJ, Hebert L (1995). The prevalence and characteristics of fibromyalgia in the general population. Arthritis Rheum.

[B4] Mease PJ, Arnold LM, Crofford LJ, Williams DA, Russell IJ, Humphrey L, Abetz L, Martin SA (2008). Identifying the clinical domains of fibromyalgia: Contributions from the clinician and patient Delphi exercises. Arthritis Rheum.

[B5] Bennett RM, Jones J, Turk DC, Matallana L (2007). An internet survey of 2,596 people with fibromyalgia. BMC Musculoskelet Disord.

[B6] Mease P, Arnold LM, Bennett R, Boonen A, Buskila D, Carville S, Chappell A, Choy E, Clauw D, Dadabhoy D, Gendreau M, Goldenberg D, Litlejohn G, Martin S, Perera P, Russell IJ, Simon L, Spaeth M, Williams D, Crofford L (2007). Fibromyalgia syndrome. J Rheumatol.

[B7] Theadom A, Cropley M, Humphrey KL (2007). Exploring the role of sleep and coping in quality of life in fibromyalgia. J Psychosom Res.

[B8] Bigatti SM, Hernandez AM, Cronan TA, Rand KL (2008). Sleep disturbances in fibromyalgia syndrome: Relationship to pain and depression. Arthritis Rheum.

[B9] White KP, Harth M (1996). An analytical review of 24 controlled clinical trials for fibromyalgia syndrome. Pain.

[B10] Harvey AG, Stinson K, Whitaker KL, Moskovitz D, Virk H (2008). The subjective meaning of sleep quality: a comparison of individuals with and without insomnia. Sleep.

[B11] Haythornthwaite JA, Hegel MT, Kerns RD (1991). Development of a sleep diary for chronic pain patients. Journal of Pain and Symptom Management.

[B12] Mease PJ, Russell IJ, Arnold LM, Florian H, Young JP, Martin SA, Sharma U (2008). A randomized, double-blind, placebo-controlled, phase III trial of pregabalin in the treatment of patients with fibromyalgia. J Rheumatol.

[B13] Arnold LM, Russell IJ, Diri EW, Duan WR, Young JP, Sharma U, Martin SA, Barrett JA, Haig G (2008). A 14-week, randomized, double-blind, placebo-controlled monotherapy trial of pregabalin in patients with fibromyalgia. J Pain.

[B14] Melzack R (1987). The short-form McGill Pain Questionnaire. Pain.

[B15] Hays R, Stewart A, Stewart A, Ware J (1992). Sleep measures. Measuring Functioning and Well-Being: the Medical Outcomes Study Approach.

[B16] Hays RD, Martin SA, Sesti AM, Spritzer KL (2005). Psychometric properties of the Medical Outcomes Study Sleep measure. Sleep Med.

[B17] Cappelleri JC, Bushmakin AG, McDermott AM, Dukes E, Sadosky A, Petrie CD, Martin S (2009). Measurement properties of the Medical Outcomes Study Sleep Scale in patients with fibromyalgia. Sleep Medicine.

[B18] Singer J (1998). Using SAS PROC MIXED to fix multilevel models, hierarchical models, and individual growth models. Journal of Educational and Behavioural Statistics.

[B19] Fleiss JL (1986). The Design and Analysis of Clinical Experiments.

[B20] Nunnally JC, Bernstein IH (1994). Psychometric Theory.

[B21] Cohen J (1988). Statistical Analysis for the Behavioral Sciences.

[B22] Abad V, Sarinas P, Guilleminault C (2008). Sleep and rheumatologic disorders. Sleep Med Rev.

[B23] Varni J, Limbers C, Burwinkle T (2007). Impaired health-related quality of life in children and adolescents with chronic conditions: a comparative analysis of 10 disease clusters and 33 disease categories/severities utilizing the PedsQL™ 4.0 Generic Core Scales. Health Qual Life Outcomes.

[B24] Eichling P, Sahni J (2005). Menopause related sleep disorders. J Clin Sleep Med.

[B25] Annemans L, Wessely S, Spaepen E, Caekelbergh K, Caubere JP, Le Lay K, Taieb C (2008). Health economic consequences related to the diagnosis of fibromyalgia syndrome. Arthritis Rheum.

[B26] Delgado J, Murali G, Goldberg R (2004). Sleep disorders in fibromyalgia. Sleep.

[B27] Khan S, Goldberg R, Haber A (2005). Sleep disorders in fibromyalgia. Sleep.

